# Genetic Screening of Plasticity Regulating Nogo-Type Signaling Genes in Migraine

**DOI:** 10.3390/brainsci10010005

**Published:** 2019-12-20

**Authors:** Gabriella Smedfors, Franziska Liesecke, Caroline Ran, Lars Olson, Tobias E. Karlsson, Andrea Carmine Belin

**Affiliations:** Karolinska Institutet, Department of Neuroscience, Biomedicum, Solnavägen 9, 17177 Stockholm, Sweden; gabriella.smedfors@ki.se (G.S.); franziska.liesecke@ki.se (F.L.); caroline.ran@ki.se (C.R.); lars.olson@ki.se (L.O.); tobias.karlsson@ki.se (T.E.K.)

**Keywords:** headache, signaling, SNP, association study, grey matter

## Abstract

Migraine is the sixth most prevalent disease in the world and a substantial number of experiments have been conducted to analyze potential differences between the migraine brain and the healthy brain. Results from these investigations point to the possibility that development and aggravation of migraine may include grey matter plasticity. Nogo-type signaling is a potent plasticity regulating system in the CNS and consists of ligands, receptors, co-receptors and modulators with a dynamic age- and activity-related expression in cortical and subcortical regions. Here we investigated a potential link between migraine and five key Nogo-type signaling genes: *RTN4*, *OMGP*, *MAG*, *RTN4R* and *LINGO1,* by screening 15 single nucleotide polymorphisms (SNPs) within these genes. In a large Swedish migraine cohort (749 migraine patients and 4032 controls), using a logistic regression with sex as covariate, we found that there was no such association. In addition, a haplotype analysis was performed which revealed three haplotype blocks. These blocks had no significant association with migraine. However, to robustly conclude that Nogo-type genotypes signaling do not influence the prevalence of migraine, further studies are encouraged.

## 1. Introduction

Migraine is the sixth most prevalent disease in the world [1]. While primarily associated with a burdening, often pulsating headache, migraine is frequently accompanied by other symptoms of altered sensory function. These symptoms vary in type, frequency and intensity between individuals. Common ones are nausea, vomiting, cutaneous allodynia, photophobia, phonophobia and osmophobia [2,3]. About one third of all migraine patients also experience the phenomenon of aura. Migraine with aura indicates transient neurological symptoms prior to the headache, often visual and/or a sensation of numbing and weakness in various parts of the body [4,5]. The migraine aura appears to be the result of cortical spreading depression which, in turn, has been associated with increased inflammation and subsequent activation of trigeminal afferents known to cause pain [6]. Migraine can be episodic (EM) with <15 attack days per month or chronic (CM) with ≥15 attack days per month [7] and patients may change from EM to CM and *vice versa* [8]. The lifetime prevalence of migraine is around 13% in Sweden [9]. Chronic migraine is reported throughout populations with a prevalence of 2% [10]. The disease affects women two to three times more often than men, and episodes in women tend to correlate to hormonal levels peaking around menstruation [11,12]. In addition to a reduced quality of life [13], individuals with migraine have an increased risk of depression [14,15], with CM patients [16] and patients with aura [17] running a higher risk than EM patients. This comorbidity is hypothesized to be caused by a shared genetic profile [18,19]. Despite intense worldwide efforts in recent decades, the underlying mechanisms causing migraine are still not fully understood, even though genome-wide association studies (GWAS) have substantially increased our knowledge of the genetic background of this complex, polygenic and multifactorial disease. The most recent GWAS, including 375,000 individuals, associated migraine to a variety of genetic loci, pointing primarily to a vascular cause but also suggesting a role for disturbances of metal ion homeostasis [20]. 

A substantial number of experiments have been conducted to analyze potential differences between the migraine brain and the healthy brain. Brain activity and morphology have been scrutinized with different techniques, such as electrophysiology, electroencephalography, magnetoencephalography, magnetic resonance imaging (MRI) and positron emission tomography [21,22,23,24,25,26,27]. Results from these investigations have identified differences between control and migraine brains with regard to resting state activity and structure of gray and white matter. Differences have been detected in the frontal lobes, corpus callosum, the limbic system, cerebellum, the brainstem and nociceptive regions, although findings are not consistent across studies [22,24,25,27,28,29,30,31,32,33,34,35,36]. In addition to regional differences, cortical thickness has been reported to differ between migraine patients and controls, and even correlate to attack frequency [37]. However, as only few longitudinal studies have been published, it remains to conclusively establish whether alteration of cortical thickness is a response to repeated migraine attacks or a predisposing condition. Furthermore, it is not known whether these structural changes may normalize with adequate treatment or spontaneous remission. The results from published investigations nevertheless indicate that disease duration and attack frequency appear to correlate with degrees of altered structures, sensitization of excitability and with altered biochemical properties. Indeed, migraine has even been proposed to possibly be a progressive brain disease [26,32,35].

**Figure 1:**
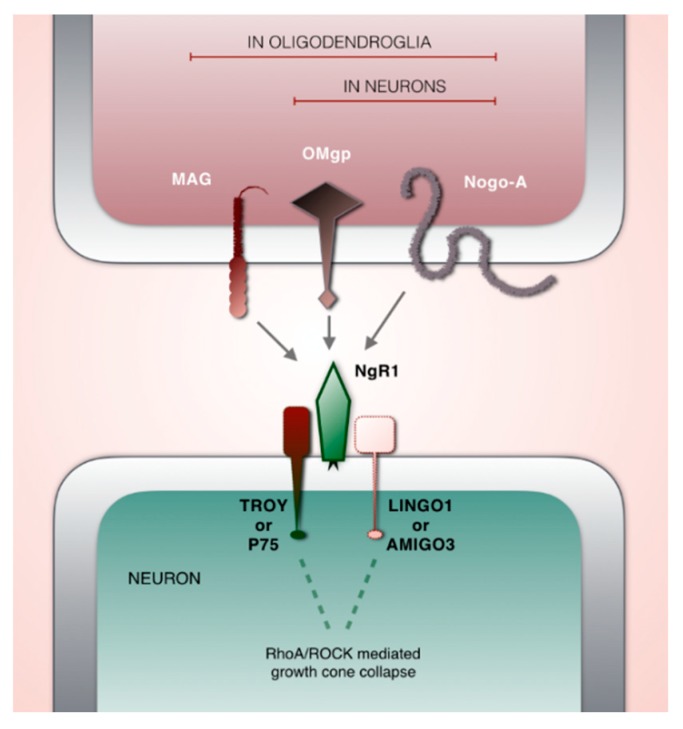
Schematic illustration of key players in Nogo-type signaling. Ligands: MAG, OMgp, Nogo-A. Key receptor: NgR1. Co-receptors: TROY or P75 and LINGO1 or AMIGO3.

The central nervous system relies on a balanced level of plasticity to adequately wire and rewire neuronal connections. Nogo type signaling [38] is known as a potent negative regulator of structural synaptic plasticity in the CNS [39,40,41,42]. It consists of ligands, receptors, co-receptors and modulators with a dynamic age- and activity-related expression in cortical and subcortical regions [43,44]. Nogo-type signaling is executed primarily via Nogo receptor 1 (NgR1) through which the ligands Nogo-A, oligodendrocyte-myelin glycoprotein (OMGP) and myelin associated glycoprotein (MAG) can signal [45,46,47,48,49]. As NgR1 is a glycophosphatidylinositol (GPI) linked receptor; the signal transmission occurs with the assistance of co-receptors TROY or P75 and LINGO1 or AMIGO3 [50,51,52,53]. Activation of NgR1 initiates an intracellular cascade through the RhoA/ROCK pathway, leading to the depolymerization of cytoskeletal actin and the collapse of axon growth cones (Figure 1) [41,48,54]. 

In epilepsy, initial seizure episodes tend to lower the threshold for additional attacks through kindling, a mechanism by which strongly activated brain pathways are thought to undergo structural synaptic plasticity. In support of this, animal modeling of electroconvulsive conditions have been shown to cause transient down regulation of NgR1 [55]. It has been suggested that migraine and affective illness may share the development of kindling with epilepsy [56]. Here, we ask if brain plasticity regulating genes involved in Nogo-type signaling are altered in migraine.

## 2. Material and Methods

Genetic information obtained from the Swedish Twin Registry was analyzed for this report, and the material has been described elsewhere [57]. All studies were performed in accordance with the Declaration of Helsinki, and procedures were carried out with written consent and adequate understanding of the test subjects. To conduct the following experiments, approval from the human subject’s ethical review board of Stockholm (reference number 2007/644-31) was received. 

The material consisted of 9897 Swedish individuals, of which 910 were classified to suffer from migraine, according to the International Classification of Headache Disorders 2nd edition ICHD-II [58]. This edition was extant at the time of the data collection and not reconciled with any changes in the newest edition (ICHD-3) affecting our study [7]. Classification was based on self-assessed questionnaires. We removed 1443 individuals from which information about migraine was not available. One twin per twin-couple was used for the association analyses. If one twin was diagnosed with migraine, this twin was kept for analysis, if both twins had migraine, one twin was randomly selected. In total, this caused removal of 4781 subjects. The cohort was checked for cryptic relatedness which removed 63 subjects, and for incorrectly assigned sex, which removed 36 subjects.

Genotyping was done on the Illumina HumanOmniExpress 12 v1.1 chip at the SNP&SEQ Technology Platform, Uppsala University. Quality control (QC) of the material included missing genotype rate per person <0.1, missing genotype rate per single nucleotide polymorphism (SNP) <0.1, minor allele frequency (MAF) <0.01 and Hardy–Weinberg equilibrium (1 × 10^−6^ for controls and 1 × 10^−10^ for cases) [59]. QC did not lead to the removal of any subjects. We found a 3.6-fold higher migraine frequency in women compared to men in our material (Table 1). This uneven, but expected, gender distribution in disease prevalence led us to use logistic regression with sex as a covariate for analysis. This controls for the risk of under- or overestimation of differences due to gender. We also performed a haplotype association test. Pairwise comparisons of markers were ignored for markers located >500 kbp apart.

The three Nogo receptor ligand genes *RTN4* (Nogo-A), *OMGP*, and *MAG*, the key Nogo receptor gene *RTN4R* (NgR1), and the co-receptor *LINGO1* were chosen for genetic analysis. To identify SNPs associated with these genes, we used the National Institute of Environmental Health Sciences software “LD TAG SNP Selection” selecting for SNPs in the central European population (CEU) [60] to match our Swedish migraine cohort. If the identified SNPs were not represented on the Illumina HumanOmni Express 12 v1.1 chip, we chose matching SNPs in high linkage disequilibrium (LD) (r^2^: 0.96–1), in order to test for indirect association. This was done for 12 SNPs via the Ensembl software (Table 2). For nine of the TAG SNPs, replacement SNPs were not available, therefore, they were excluded from the study (Table S1). The remaining TAG SNPs and replacement SNPs were tested for high LD to exclude SNP-pairs with r^2^ > 0.2 [61]. This step led to the rejection of 17 additional SNPs, leaving 15 SNPs for further analysis.

Genetic analyses were made with PLINK versions 1.07 and 1.9 [62,63]. Power calculations were made online with help of the Genetic Association Study Power Calculator [64] with MAF reference-values from National Center for Biotechnology Information [65], choosing the Northern Sweden population (Table 3). Haplotype analysis was made with the Haploview software from the Broad institute [66]. For graphical and further statistical analysis, we used R and RStudio version 1.1.456 [67,68].

## 3. Results

Nogo-type signaling involves a broad number of receptors, ligands, co-receptors and modulators. To increase the power of our study, we chose to analyze five key genes: *RTN4*, *RTN4R*, *LINGO1*, *OMGP,* and *MAG*. After QC (see methods), our cohort consisted of 4781 individuals, 749 cases and 4032 controls, and we selected 15 SNPs in, or in proximity to, these genes for the association analysis (Table 3). When we used the association analysis in the form of a logistic regression analysis with sex as a covariate due to the expected 3.6 times higher prevalence of migraine among females in our cohort, we did not reveal an association between any of these 15 SNPs and migraine.

As haplotypes are considered more valuable to predict genetic correlations with disease outcome than single SNPs alone [69], we also performed a haplotype analysis. The analysis revealed three haplotype blocks (Table 4). These blocks had no significant association with migraine. 

**Figure 2:**
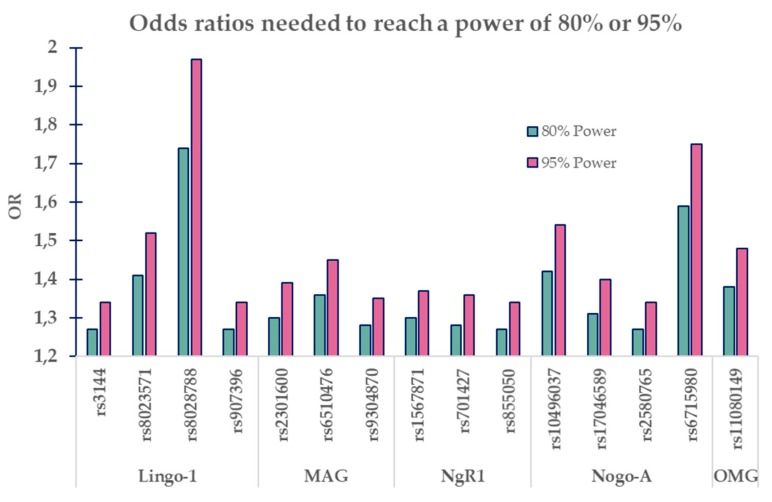
Graph illustrating needed odds ratios (ORs) for SNP’s minor allele frequencies to reach a power of 80% or 95%.

To estimate what effect-size we would need to be able to reach a power of 80% or 95%, we performed power calculations based on the MAF of each SNP in our population (Figure 2). The two levels of power were selected with the rationale that this was a study of collected data from a very specific population. Hence, a replication study where each replicate would decrease the risk of missing an actual effect is hard to obtain. This is based on the commonly chosen power of 80% which would declare 1/5 of the SNPs having a significant association as non-significant.

## 4. Discussion

Migraine carries dire consequences for the suffering patients, including socio-economic misfortune. In the United States alone, the societal direct and indirect costs were estimated to be $36 billion in 2016 [70]. Progress in the understanding of migraine pathophysiology is central to discover better therapies. 

It has repeatedly been demonstrated how the migraine brain differs morphologically from the healthy brain in several brain regions. Moreover, these alterations appear to progress over time and with attack frequency [25,28,31,32,33,34,35]. However, there may well be alterations at the level of structural synaptic plasticity that cannot be detected in vivo in humans with available methods. Here, we investigated a potential association between a key plasticity-regulating system in the CNS—Nogo-type signaling—and migraine. 

Microstructural alterations associated with Nogo-type signaling cannot be investigated with MRI and related methods, since voxel sizes trespass the size of dendrites and dendritic spines [32,71]. Since satisfying animal models of migraine are lacking, we chose a genetic approach to investigate Nogo-type signaling in migraine. We looked at the frequency of 15 SNPs from five genes primarily associated with Nogo-type signaling; the ligands *RTN4*, *OMGP* and *MAG*, the key receptor *RTN4R*, and the co-receptor *LINGO1*. The most recent GWAS of migraine identified 38 suspected loci [20], none of them a part of Nogo-type signaling. However, as GWAS handles huge numbers of genetic targets, it not only suffers risk of detecting false-positive associations, its need for profound correction for multiple testing may result in loss of smaller effects.

We analyzed SNPs related to Nogo-type signaling in a cohort of the Swedish Twin Registry, consisting of 749 migraine cases and 4032 controls where the migraine frequency was 3.6 times higher among women. When analyzing the association of the Nogo SNPs with sex as covariate, we found no significant association of any of the selected genes to migraine. Our findings decrease the likelihood of altered Nogo signaling being a risk factor for migraine but does not exclude this possibility.

Migraine patients differ regarding age of onset, occurrence of aura, frequency of attacks, attack-causing stimuli, and attack intensity. Furthermore, different patients respond to different medications. For some patients, over the counter painkillers can abort an attack while for other patients, extensive polypharmacological treatments may not be sufficient. These differences in response to medication presumably reflect different genetic profiles [72]. A limiting aspect of the current study is that information about these variations among the migraine patients was lacking.

ICHD defines chronic migraine as having fifteen or more attack days per month. However, already in patients with episodic migraine, suffering from three attack days (and more, but less than 15) per month have been shown to be associated with structural differences [32]. The fact that structural changes are noticeable at a five times lower frequency than the criteria of the chronic state increases the incentive for a more aggressive therapy to alleviate symptoms by reducing attack frequency early on, also in episodic migraine patients.

Our results should also be viewed in light of the power analysis which revealed that with our sample size, we would need odds ratios extending the ORs acquired in this report, and the ORs in the last migraine GWAS [20]. Our ORs were 0.97–1.17 and the SNPs positively associated with migraine in the migraine GWAS had ORs ranges of 0.88–1.11. Our power calculation pointed out that for 80% power, our SNPs would need ORs between 1.27 and 1.74 and for 95% power, ORs of 1.34–1.97. Thus, this study defines a theoretical upper level of how strongly these SNPs could influence the prevalence of migraine. When more studies emerge, this initial estimated effect can be improved, and a more exact effect/non-effect can be established. 

## 5. Conclusions

Nogo-type signaling comprises a potent negative regulator system for structural synaptic plasticity. We investigated a potential link between Nogo-type signaling and migraine based on the frequency of 15 SNPs associated with five genes involved in Nogo-type signaling in 4781 individuals, of which 749 had migraine, in a Swedish cohort. We did not detect an association of any of the 15 SNPs with migraine. Our findings suggest that altered Nogo-type signaling does not strongly affect the pathophysiology of migraine.

## Figures and Tables

**Table 1:** Distribution of individuals for analysis.

	Controls	Cases	Total	Migraine Frequency
Men (%)	2138 (53.0)	162 (21.6)	2300	7.0%
Women (%)	1894 (47.0)	587 (78.4)	2481	23.7%
Total	4032	749	4781	15.7%

**Table 2:** Nogo-type signaling single nucleotide polymorphism (SNP) selection for association analysis.

Chr	Gene Symbol	TAG SNPs of Interest	Replacement SNP	After Exclusion of SNPs in LD with r^2^ > 0.2
2	RTN4	rs6545465	rs17046589	rs17046589
2	RTN4	rs7562292	rs6545466	-
2	RTN4	rs10084445	rs6715980	rs6715980
2	RTN4	rs7584386	rs7584354	-
2	RTN4	rs2580765	-	rs2580765
2	RTN4	rs17046594	rs17046570	-
2	RTN4	rs3198123	-	-
2	RTN4	rs2580769	-	-
2	RTN4	rs2588517	-	-
2	RTN4	rs2588519	-	-
2	RTN4	rs2864052	-	-
2	RTN4	rs10496037	-	rs10496037
2	RTN4	rs2920898	-	-
15	LINGO1	rs907395	rs907396	rs907396
15	LINGO1	rs8024724	rs8023571	rs8023571
15	LINGO1	rs3743481	-	-
15	LINGO1	rs7162113	-	-
15	LINGO1	rs3144	-	rs3144
15	LINGO1	rs1877298	rs8028788	rs8028788
17	OMG	rs11080149	-	rs11080149
19	MAG	rs12461927	rs720308	-
19	MAG	rs12185485	rs3746248	-
19	MAG	rs10414549	-	-
19	MAG	rs9304870	-	rs9304870
19	MAG	rs6510476	-	rs6510476
19	MAG	rs2301600	-	rs2301600
19	MAG	rs10411883	rs11669734	-
22	RTN4R	rs854971	rs701427	rs701427
22	RTN4R	rs1567871	-	rs1567871
22	RTN4R	rs855050	-	rs855050
22	RTN4R	rs1807466	-	-
22	RTN4R	rs887765	-	-

Table of 32 SNPs associated with five Nogo-type signaling genes and their replacement SNPs if original SNPs were not available on the Illumina OmniExpress chip. SNPs in LD with r2 > 0.2 were excluded. SNPs in the rightmost column were used for further association analysis with migraine in the Swedish twin cohort. Chr = Chromosome, SNP = Single Nucleotide Polymorphism, LD = Linkage Disequilibrium.

**Table 3:** Fifteen SNPs associated with Nogo-type signaling investigated for association to migraine in a Swedish cohort.

Gene	SNP	Function	Minor Allele	MAF NCBI	MAF Cases	MAF Controls	OR (95% CI)	*P*-Value	Corrected *P*-Value
RTN4	rs2580765	Intron	C	0.46	0.46	0.43	1.09 (0.97–1.22)	0.14	1
RTN4	rs6715980	Intron	A	0.06	0.07	0.07	1.04 (0.83–1.29)	0.76	1
RTN4	rs17046589	Intron	G	0.22	0.18	0.18	1.003 (0.87–1.16)	0.96	1
RTN4	rs10496037	Intron	T	0.11	0.12	0.11	1.08 (0.91–1.29)	0.36	1
LINGO1	rs3144	3’ UTR region	C	0.40	0.37	0.37	0.97 (0.86–1.09)	0.56	1
LINGO1	rs907396	Intron	G	0.40	0.40	0.38	1.1 (0.98–1.24)	0.11	1
LINGO1	rs8023571	Intron	T	0.12	0.12	0.12	1.02 (0.86–1.22)	0.79	1
LINGO1	rs8028788	Intron	C	0.04	0.05	0.04	1.17 (0.91–1.52)	0.23	1
OMGP	rs11080149	Coding	T	0.14	0.17	0.15	1.08 (0.92–1.25)	0.35	1
MAG	rs6510476	Intron	G	0.16	0.18	0.18	1.01 (0.87–1.17)	0.92	1
MAG	rs2301600	Coding	T	0.24	0.25	0.23	1.07 (0.94–1.22)	0.33	1
MAG	rs9304870	Intron	G	0.33	0.38	0.38	1.03 (0.91–1.15)	0.66	1
RTN4R	rs701427	Intron	A	0.31	0.32	0.34	0.93 (0.83–1.05)	0.26	1
RTN4R	rs1567871	Intron	T	0.26	0.25	0.25	1.0 (0.88–1.14)	1.00	1
RTN4R	rs855050	Intron	G	0.49	0.51	0.50	1.04 (0.93–1.17)	0.47	1

Chr = Chromosome, SNP = Single Nucleotide Polymorphism, 3’ UTR = three prime untranslated region, MAF = Minor Allele Frequency, OR = Odds Ratio, CI = confidence interval, *P*-values = α 0.05, Corrected *P*-value = Bonferroni correction based on α/15 (nr of SNPs).

**Table 4:** Identified haplotypes in three genes associated to Nogo-type signaling were not associated to migraine.

	Block	Haplotype	Frequency	Case-Control Frequencies	*P*-Value
*LINGO1*	rs907396 rs8023571	CC	0.41	0.43/0.41	0.17
		AC	0.34	0.32/0.34	0.21
		CT	0.25	0.25/0.25	0.83
*MAG*	rs6510476 rs2301600	AC	0.59	0.58/0.59	0.23
		AT	0.23	0.25/0.23	0.18
		GC	0.18	0.18/0.18	0.96
*RTN4R*	rs701427 rs1567871	TC	0.50	0.49/0.50	0.26
		GC	0.38	0.39/0.38	0.26
		TT	0.12	0.12/0.12	0.89

## Supplementary Materials

The following are available online at https://www.mdpi.com/2076-3425/10/1/5/s1, Table S1: Excluded SNPs due to absence on the genotyping chip and lack of replacement SNPs in high LD on the current chip.
